# The Computer Simulation of Therapy with the NMDA Antagonist in Excitotoxic Neurodegeneration in an Alzheimer’s Disease-like Pathology

**DOI:** 10.3390/jcm11071858

**Published:** 2022-03-27

**Authors:** Dariusz Świetlik, Aida Kusiak, Marta Krasny, Jacek Białowąs

**Affiliations:** 1 Division of Biostatistics and Neural Networks, Medical University of Gdansk, Debinki 1, 80-211 Gdansk, Poland; 2 Department of Periodontology and Oral Mucosa Diseases, Medical University of Gdansk, Debowa 1a, 80-204 Gdansk, Poland; aida.kusiak@gumed.edu.pl; 3 Medicare Dental Clinic, Popieluszki 17a/102, 01-595 Warsaw, Poland; mkrasny@op.pl; 4 Division of Anatomy and Neurobiology, Medical University of Gdansk, Debinki 1, 80-211 Gdansk, Poland; jacekwb@gumed.edu.pl

**Keywords:** NMDA antagonists, memantine, Alzheimer’s disease, neural networks, computer simulation, virtual therapy

## Abstract

(1) Background: The use of uncompetitive N-methyl-D-aspartate (NMDA) receptor antagonists results in neuroprotective benefits in patients with moderate to severe Alzheimer’s disease. In this study, we demonstrated mathematical and computer modelling of the excitotoxicity phenomenon and performed virtual memantine therapy. (2) Methods: A computer simulation environment of the N-methyl-D-aspartate receptor combining biological mechanisms of channel activation by means of excessive extracellular glutamic acid concentration in three models of excitotoxicity severity. The simulation model is based on sliding register tables, where each table is associated with corresponding synaptic inputs. Modelling of the increase in extracellular glutamate concentration, through over-stimulation of NMDA receptors and exacerbation of excitotoxicity, is performed by gradually increasing the parameters of phenomenological events by the power function. Pathological models were virtually treated with 3–30 µM doses of memantine compared to controls. (3) Results: The virtual therapy results of memantine at doses of 3–30 µM in the pathological models of excitotoxicity severity show statistically significant neuroprotective benefits in AD patients with moderate severity, 1.25 (95% CI, 1.18–1.32) vs. 1.76 (95% CI, 1.71–1.80) vs. 1.53 (95% CI, 1.48–1.59), (*p* < 0.001), to severe, 1.32 (95% CI, 1.12–1.53) vs. 1.77 (95% CI, 1.72–1.82) vs. 1.73 (95% CI, 1.68–1.79), (*p* < 0.001), in the area of effects on memory. A statistically significant benefit of memantine was demonstrated for all neuronal parameters in pathological models. In the mild severity model, a statistically significant increase in frequency was obtained relative to virtual memantine treatment with a dose of 3 µM, which was 23.5 Hz (95% CI, 15.5–28.4) vs. 38.8 Hz (95% CI, 34.0–43.6), (*p* < 0.0001). In the intermediate excitotoxicity severity model, a statistically significant increase in frequency was obtained relative to virtual memantine therapy with a 3 µM dose of 26.0 Hz (95% CI, 15.7–36.2) vs. 39.0 Hz (95% CI, 34.2–43.8) and a 10 µM dose of 26.0 Hz (95% CI, 15.7–36.2) vs. 30.9 Hz (95% CI, 26.4–35.4), (*p* < 0.0001). A statistically significant increase in frequency was obtained in the advanced excitotoxicity severity model as in the medium. (4) Conclusions: The NMDA antagonist memantine causes neuroprotective benefits in patients with moderate to severe AD. One of the most important benefits of memantine is the improvement of cognitive function and beneficial effects on memory. On the other hand, memantine provides only symptomatic and temporary support for AD patients. Memantine is prescribed in the US and Europe if a patient has moderate to severe AD. Memantine has also been approved for mild to moderate AD patients. However, its very modest effect provides motivation for further research into new drugs in AD. We are the first to present a mathematical model of the NMDA receptor that allows the simulation of excitotoxicity and virtual memantine therapy.

## 1. Introduction

According to the World Health Organization (WHO), the number of patients with Alzheimer’s disease (AD) will reach 65 million in 2030 and 115 million in 2050. In Poland, the number of patients will triple by 2050 to approximately one million [1]. AD is a highly progressive neurodegenerative disease, and clinical manifestations include the impairment of memory and other cognitive functions [2,3,4,5]. The brains of AD patients show transmission deficits in most neurotransmitter systems, with glutamatergic dysfunction being the most prominent. Glutamate plays an important role in physiological processes of learning and memory trace formation, mainly in the mechanism of the so-called long-term potentiation (LTP) phenomenon [6,7].

Excitotoxicity plays a significant role in the development and progression of AD and other neurodegenerative disorders [8,9]. A disruption of calcium ion homeostasis acts excitotoxically, leading to the process of neurodegeneration in AD and contributing to nerve cell death [10,11,12]. Excitotoxic nerve cell damage occurs mainly due to the overstimulation of N-methyl-D-aspartate (NMDA). The hypothesis formulated by Parsons and Danysz assumes that elevated glutamate concentration (though not high enough to lead to neuronal death in the mechanism of excitotoxicity) leading to continuous but irregular stimulation of NMDA receptors makes it difficult for the actual calcium signal carrying the relevant information to “get through” the “glutamate noise”, which prevents the formation of a memory trace [13].

The use of uncompetitive NMDA receptor antagonists in preclinical studies and clinical trials has shown that memantine has the best pharmacological profile and best tolerability [14,15,16,17,18]. Memantine received European marketing authorization in 2002 and US Food and Drug Administration (FDA) approval in 2003. It is mainly used for the treatment of moderate to severe forms of AD [15]. Memantine has shown positive effects on improving cognitive abilities and reducing neurodegeneration in AD patients [16,19].

Computer models of neurons [20] and neural networks [21,22] are one of the methods of understanding the functioning of the nervous system, which we cannot study under natural conditions due to the limitation of modern research methods. Computer models of synaptic decay in the hippocampus for different phases of synapse loss [23] are helpful in understanding the process of neurodegeneration in Alzheimer’s disease. In contrast, other simulation studies show that inducing gamma oscillations in the hippocampus can ameliorate the pathology associated with Alzheimer’s disease [24]. The artificial neural networks have been successfully used in supporting the diagnosis of AD based on cerebral perfusion single-photon emission computed tomography (SPECT) data and in nuclear medicine [25,26].

In silico research allows us, among other things, to understand the cellular mechanisms underlying laminopathy [27]. Molecular dynamics simulations have produced substantial new data in a variety of in silico investigations [27,28,29,30,31,32,33].

In this study, a computer simulation environment of α-amino-3-hydroxy-5-methyl-4-isoxazolepropionic (AMPA) and NMDA receptors was created for virtual excitotoxicity therapy with the NMDA receptor antagonist-memantine using the mathematical formalism from previous simulation studies [20,21,22,23,24]. Memantine inhibits NMDA receptor currents in a concentration-dependent manner. The virtual therapy was simulated by a change in threshold for the removal of the Mg^2+^ ion block for NMDA channels. The model included (1) the biological mechanism of AMPA and NMDA receptor function, (2) simulations of glutamate release inside the synaptic gap following presynaptic stimulation, (3) the mechanism of excitotoxicity, (4) simulations of memantine treatment at three concentrations: 3, 10 and 30 µM, due to the pattern of long-term potentiation (LTP). The results of the in silico virtual therapy study show new possibilities for bioinformatics, in which real biological processes can be simulated in a virtual environment.

In Section 2, we introduce mathematical models: Synaptic properties in Section 2.1, Long-Term Potentiation (LTP) and LTP Duration in Section 2.2, and Excitotoxicity Model in Section 2.3. In Section 2.4, we present virtual therapies with the NMDA antagonist of three concentrations of memantine: 3, 10 and 30 µM. Additionally, in Section 2.5, we describe the statistical analysis and the used software. In Section 3, we present our results; NMDA receptor activity under physiological and pathological conditions and virtual therapy with memantine is included in Section 3.1. In simulations comparing the control model with pathological models (moderate to severe Alzheimer’s disease) and the virtual therapy results of memantine at doses of 3–30 µM, the following parameters were used: interspike interval (ISI), excitatory postsynaptic potential (EPSP) and frequency, as described in Section 3.2. In Section 3.3, we discuss LTP and LTP duration. In Section 4, we fully discuss our results. Section 5 summarizes the conclusions. In Section 6, we discuss study limitations.

The computer simulation of therapy with the NMDA antagonist is used in our study to examine the efficacy of medication in moderate to severe Alzheimer’s disease.

## 2. Methods

**Figure 1:**
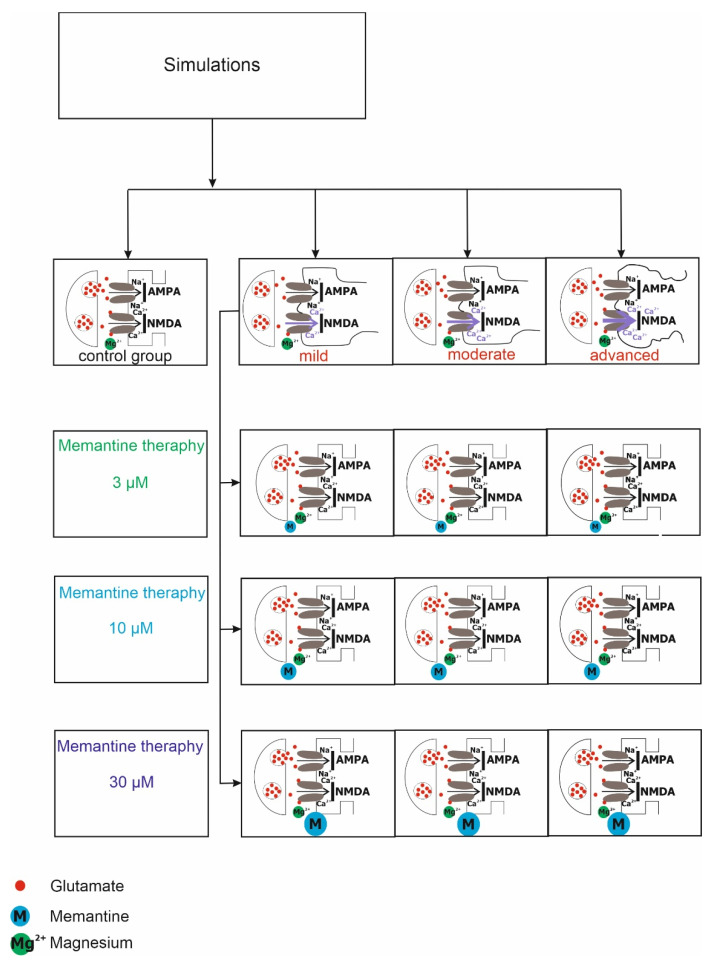
The diagram of the simulation. Computer simulations were performed for the control group and degrees of excitotoxicity: mild, moderate and advanced. Simulations of memantine treatment at three concentrations: 3, 10 and 30 µM for AD groups.

This chapter will describe in detail the individual modules that make up the computer simulation model. Each module is responsible for a different part of the overall synaptic transmission process. The simulation model uses the formalism from previous studies [20,21,22,23,24] and a simplified demo version of the simulation model available on ModelDB-private model (https://senselab.med.yale.edu/modeldb)—accessed data: 1 June 2019; or (https://medinf.gumed.edu.pl/383.html)—accessed data: 1 September 2017. The diagram of the simulation is shown in Figure 1.

### 2.1. Synaptic Properties

The simulation model is based on sliding register tables, where each table is associated with corresponding synaptic inputs. The excitatory synaptic inputs have two registers, E(t), associated with the glutamine receptor AMPA, and the memory register, M(t), associated with the glutamine receptor NMDA. The synaptic function SF(t) comes in two forms, SF_AMPA_(t) and SF_NMDA_(t), according to the two registers, E(t) and M(t), respectively (Equation (2.1), Świetlik, 2018 [22]). At the beginning of each simulation, all values in the E(t) and M(t) tables in the registers were filled with the residual potential value ReP = −80 mV. When an action potential arrived at a given input, the synaptic function SF(t) wrote the calculated values into the corresponding register.

### 2.2. Long-Term Potentiation (LTP) and LTP Duration

Physiological stimulation of the NMDA receptor is essential for neuroplasticity and LTP processes [34]. The issue of modelling the plasticity of biological synapses is very difficult, but our algorithm mimics quite accurately the biologically most important process of long-term potentiation (LTP), and at the same time takes into account the process of forgetting, i.e., the return of the weight of a given synapse to its initial state in the absence of maintenance mechanisms [20,21,22,23,24]. We used a function that calculates the current synapse weight magnitude and models the biological process of long-term potentiation and synaptic efficacy as an exponent from the LTP function. If the summarized potential in compartment k is greater than the threshold for the removal of the Mg^2+^ ion block for NMDA channels, then

(1) $$\mathrm{LTP}=1+\ln\frac{C_{k}+1}{6\;\mathrm{clog}},$$

where C_k_ is the time of memory for compartment k and parameter clog = 2.3026. LTP induction happens when there is an action potential on the excitatory input and open NMDA channels due to adequate depolarization of the postsynaptic region. The amount of calcium ions influenced throughout the opened NMDA channels influences the initiation of the cascade of physiological processes that lead to LTP.

We used a power function to model the phenomenological event:

power = powerA (M − ReP),(2)

where powerA = 9 is a parameter and M is the actual value of synaptic function SF(t) for excitatory postsynaptic potentials. LTP duration:

LTP duration = exp (power) − 1,(3)

Clog and powerA are numerical parameters that allow the strength and LTP duration to be constrained to biologically reasonable values in each simulation. Equations (1)–(3) use formalism from previous studies [20,21,22,23,24].

### 2.3. Excitotoxicity Model

Excessively high concentrations of glutamate can lead to sustained stimulation of NMDA receptors and a massive influx of Ca^2+^ ions into the cell [28]. The initiation of the cascade of biochemical reactions leading to LTP depends on the amount of Ca^2+^ calcium ions entering the cell through open NMDA channels. This process is modelled by the “power” function Equation (2). In contrast, glutamate overactivity (excitotoxicity) results in neuronal damage and overactivation leads to increased energy demand. Modelling of the increase in extracellular glutamate concentration, through over-stimulation of NMDA receptors and exacerbation of excitotoxicity, is performed by gradually increasing the “powerA” parameter in Equation (2). In the control model and in the intensity of the excitotoxicity phenomenon, the following values were taken, respectively: 9, 56.7, 63 and 135. The study groups related to the severity of excitotoxicity were divided into mild, moderate and very advanced.

### 2.4. Virtual Therapy with the NMDA Antagonist Memantine

In situations of prolonged, pathological NMDA receptor activation and associated channel opening, memantine blocks the NMDA receptor and prevents excitotoxicity from occurring. Memantine thus prevents pathological activation of the receptor without blocking the possibility of physiological activation. In this way, memantine increases the electrophysiological signal-to-noise ratio. Thus, the detection of a “meaningful” stimulus becomes possible and physiological LTP, responsible for learning and memory, can be restored [35,36]. The virtual therapy was carried out at three concentrations of memantine: 3, 10 and 30 µM. The computer modelling used studies that showed that memantine inhibits NMDA receptor currents in a concentration-dependent manner with IC50 values (concentration causing 50% inhibition) in the range of 0.5–10 µM at hyperpolarized membrane potentials (−30 to −70 mV) [37,38,39,40,41].

### 2.5. Statistical Analysis

Statistical analysis was performed using software from TIBCO Software Inc., (Palo Alto, CA, USA; 2017), Statistica (data analysis software system), version 13. http://statistica.io (accessed data 1 September 2017) [42]. Shapiro–Wilk and Leven (Brown-Forsythe) tests of concordance were used to check the origin from the population with normal distribution and homogeneity of variance, respectively. The significance of differences between study groups and controls was checked by F-test (ANOVA) or Kruskal–Wallis test. When statistically significant differences between groups were obtained, Tukey’s and Dunn’s post hoc tests were used, respectively. In all calculations, α = 0.05 was taken as the significance level.

## 3. Results

In Section 3, we present our results; NMDA receptor activity under physiological and pathological conditions and virtual therapy with memantine is included in Section 3.1. In simulations comparing the control model with pathological models (moderate to severe Alzheimer’s disease) and virtual therapy results of memantine at doses of 3–30 µM, the following parameters were used: ISI, EPSP and frequency, as described in Section 3.2 Neuronal Parameters. In Section 3.3, we discuss LTP and LTP duration.

### 3.1. NMDA Receptor Activity under Physiological, Pathological Conditions and Virtual Therapy with Memantine

**Figure 2:**
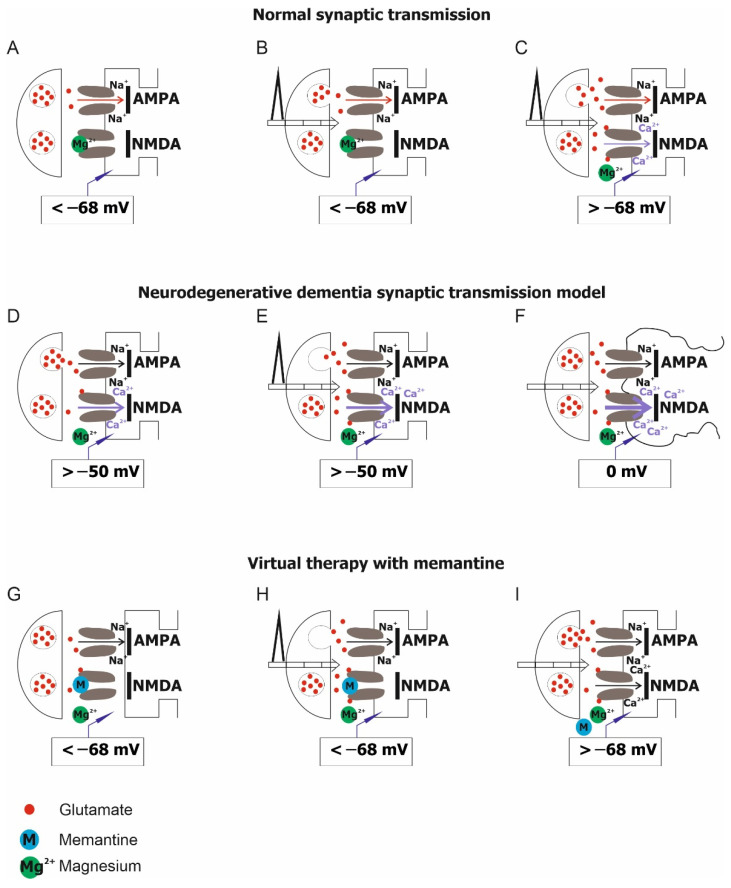
NMDA receptor activity under physiological conditions (normal synaptic transmission), pathological conditions (neurodegenerative dementia synaptic transmission) and virtual therapy with memantine. (**A**) Current flow through NMDAR channels is blocked by external Mg^2+^ ions at resting membrane potentials. (**B**,**C**) The stimulus-induced activation of the receptor leads to the unblocking of the channel and the influx of Ca^2+^ ions inside the cell. (**D**,**E**) Activation of the receptor caused by neurotoxic factors results in the release of Mg^2+^ and an uncontrolled influx of Ca^2+^ into the cell. (**F**) Potentiometer at 0 mV indicates cell death. (**G**) Channel blocked with memantine at rest. (**H**) Memantine is able to block the channel for the flow of Ca^2+^ ions despite the increased concentration of glutamic acid and the increased depolarization potential. (**I**) The depolarization caused by a strong stimulus is sufficient to remove the blockade of the memantine channel and allow the influx of calcium ions into the cell.

Under physiological conditions, NMDA receptors very often do not participate in the process of standard excitatory transmission because they are blocked by Mg^2+^ ions and have unique properties. At the hyperpolarization potential of the cell membrane of about −68 mV, the negative intracellular electric field supports the entry of Mg^2+^ ions into the NMDA receptor ionophore. Consequently, the channel is blocked and impermeable to ions (Figure 2A).

The stimulation of the synapse by glutamic acid, with AMPA receptor involvement, will result in an influx of Na^+^ ions in particular (Figure 2B). However, when glutamic acid stimulates the synapse very strongly, also via the AMPA receptor, Mg^2+^ ions are released from the NMDA receptor ionophore, ions (mainly Na+ and Ca^2+^) flow into the cell via the unblocked channel, and postsynaptic activation occurs (Figure 2C).

In pathological conditions, the NMDA receptor is activated by an excessive concentration of extracellular glutamic acid. This condition occurs not only under the influence of neurodegenerative factors, but also during prolonged seizures and as a result of neuronal destruction or death, e.g., as a result of ischemia. In such a situation, the membrane potential increases, becoming more positive, and this causes the release of magnesium ions that block the NMDA receptor (Figure 2D). The increased concentration of extracellular glutamic acid leads to an intense flow of calcium Ca^2+^ ions through the unblocked NMDA channel (Figure 2E) and the consequent electrophysiological noise that prevents the detection of ‘meaningful’ stimuli that normally, via the NMDA receptor-mediated influx of Ca^2+^ ions into the cell, initiating the process underlying LTP. The occurrence of this process for long enough leads to cell damage or death (Figure 2F).

Blocking of the NMD receptor ionophore by memantine is potential-dependent (Figure 2G). A stronger depolarising stimulus is required to release memantine and unblock the channel than for Mg^2+^-mediated blockade. Memantine reduces excessive calcium ion flow through NMDA receptors without blocking the flow induced by the physio-logical stimulus (Figure 2H). When a strong stimulus induces a large enough depolarisation of the postsynaptic membrane, memantine is released and the channel becomes permeable to calcium ions (Figure 2I). In this way, memantine increases the electro-physiological signal-to-noise ratio. This makes the detection of a ‘meaningful’ stimulus possible, and physiological LTP, responsible for learning and memory, can be restored.

### 3.2. Neuronal Parameters

The following neuronal parameters were statistically analyzed: interspike interval (ISI) (the interspike interval is the time between subsequent action potentials), number of spikes, excitatory postsynaptic potential (EPSP) and frequency. Additionally, all analyses were performed for three periods, before LTP induction, during 100 Hz stimulation and after LTP induction.

#### 3.2.1. ISI, EPSP and Frequency

##### Control Group

**Figure 3:**
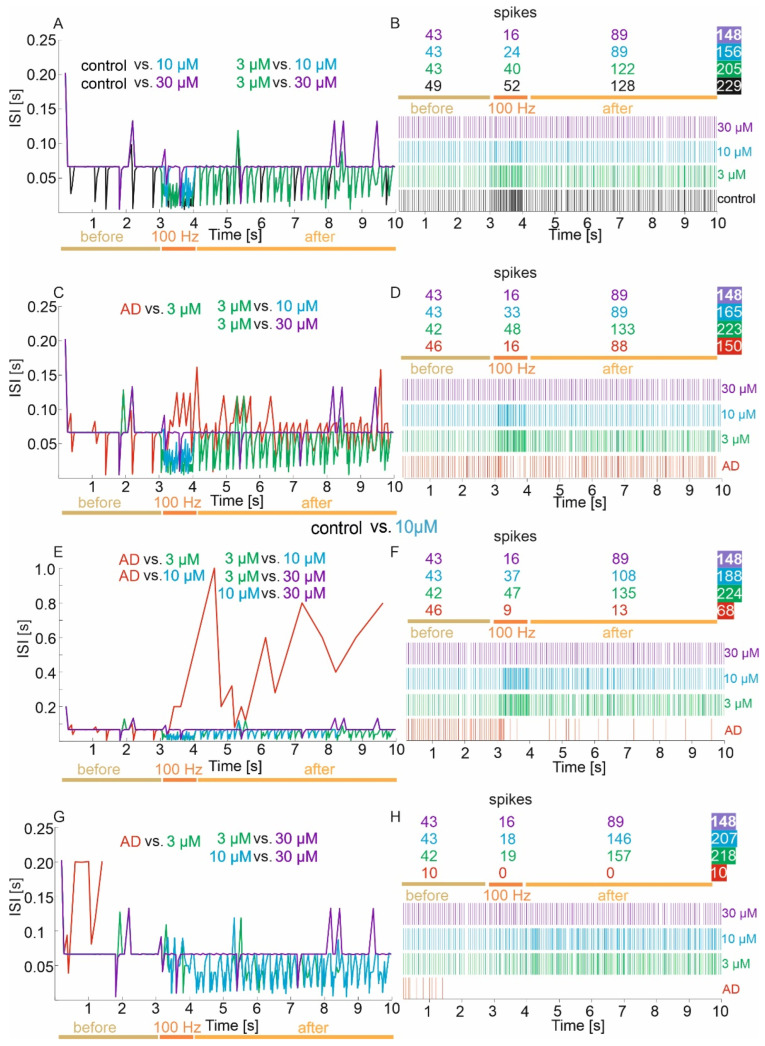
Comparison of ISI in virtual therapy treatment of memantine treatment at three concentrations: 3, 10 and 30 µM and output spike trains. (**A**,**B**) Control group, (**C**,**D**) mild degree of excitotoxicity, (**E**,**F**) moderate degree of excitotoxicity, and (**G**,**H**) advanced degree of excitotoxicity.

The mean values and 95% CI for ISI in the control group, memantine concentrations of 3, 10 and 30 µM are 0.044 (95% CI, 0.040–0.047) vs. 0.049 (95% CI, 0.045–0.052) vs. 0.064 (95% CI, 0.061–0.067) vs. 0.067 (95% CI, 0.064–0.070), respectively. There were statistically significant ISI differences between groups (*p* < 0.001). Exact post hoc tests showed that the ISI value was significantly lower in the control group relative to concentrations of 10 µM (*p* < 0.000001) and 30 µM (*p* < 0.000001). Furthermore, ISI was significantly lower in the 3 µM concentration group relative to the 10 µM (*p* < 0.000001) and 30 µM (*p* < 0.000001) concentrations. There was no statistically significant difference in ISI of the control compared to the 3 µM concentration (*p* = 0.3442) (Figure 3A).

Detailed statistical analysis of ISI against groups in the period before LTP induction showed no statistically significant differences (*p* > 0.05), while statistically significant differences (*p* < 0.0001) were obtained in the induction period and after LTP. During the 100 Hz induction period, it was obtained that the ISI value was significantly lower in the control group compared to the 10 µM (*p* = 0.0005) and 30 µM (*p* < 0.000001) concentrations. In addition, ISI was significantly lower in the 3 µM concentration group relative to the 30 µM concentration (*p* < 0.000001). There was no statistically significant difference in ISI of the control compared to the 3 µM concentration (*p* = 0.5129), the 10 µM concentration against the 3 µM (*p* = 0.1079) and 30 µM (*p* = 0.1147). In the period after the 100 Hz induction, the results were identical to the total value (Figure 3A).

**Figure 4:**
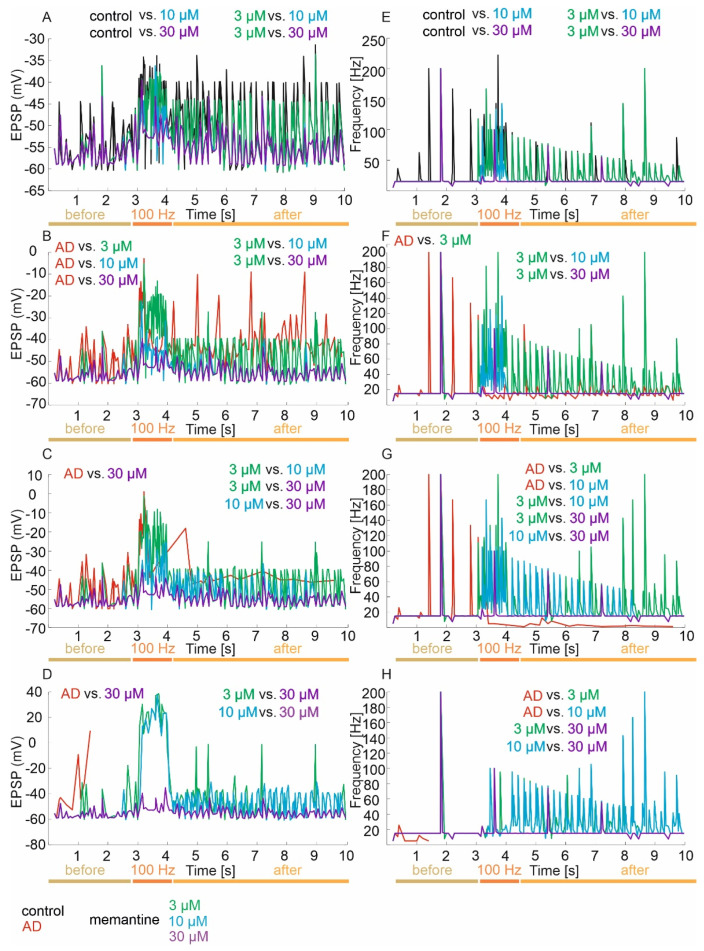
Comparison of EPSP and frequency in virtual therapy treatment of memantine treatment at three concentrations: 3 µM, 10 µM and 30 µM. (**A**,**E**) Control group, (**B**,**F**) mild degree of excitotoxicity, (**C**,**G**) moderate degree of excitotoxicity, and (**D**,**H**) advanced degree of excitotoxicity.

The mean values and 95% CI for EPSP in the control group and memantine concentrations of 3, 10 and 30 µM are −49.4 mV (95% CI, −50.4–48.3) vs. −51.3 mV (95% CI, −52.2–50.4) vs. −54.4 mV (95% CI, −55.1–53.6) vs. −54.9 mV (95% CI, −55.5–54.2), respectively. There were statistically significant differences in EPSP versus groups (*p* < 0.0001). Exact post hoc tests showed that EPSP was significantly higher in the control group relative to concentrations of 10 µM (*p* < 0.000001) and 30 µM (*p* < 0.000001). In addition, EPSP was significantly higher in the 3 µM concentration group relative to the 10 µM (*p* = 0.0011) and 30 µM (*p* = 0.0001) concentrations. There was no statistically significant difference in EPSP of the control compared to the 3 µM concentration (*p* = 0.1834) and the 10 µM concentration compared to the 30 µM concentration (*p* = 1.00) (Figure 4A).

Detailed statistical analysis of EPSP against groups in the period before LTP induction showed no statistically significant differences (*p* = 0.5481), while statistically significant differences (*p* < 0.0001) were obtained in the induction period and after LTP. During the 100 Hz induction period, it was obtained that EPSP was significantly higher in the control group relative to the 10 µM (*p* = 0.0018) and 30 µM (*p* = 0.0002) concentrations. Furthermore, EPSP was significantly higher in the 3 µM concentration group relative to the 10 µM (*p* = 0.0370) and 30 µM (*p* = 0.0039) concentrations. There was no statistically significant difference in the EPSP of the control compared to the 3 µM concentration (*p* = 1.00), or for the 10 µM concentration compared to the 30 µM concentration (*p* = 1.00). In the post-induction 100 Hz period, the results were similar to the total value—namely, the control group had significantly higher EPSP compared to 10 µM (*p* < 0.000001) and 30 µM (*p* < 0.000001) concentrations and EPSP was significantly higher in the 3 µM concentration group compared to the 10 µM (*p* = 0.0077) and 30 µM (*p* = 0.0087) concentrations. There was no statistically significant difference in EPSP of the control compared to the 3 µM concentration (*p* = 0.6027) and the 10 µM concentration compared to the 30 µM concentration (*p* = 1.00) (Figure 4A).

The mean values and 95% CI for frequency in the control group, plus memantine concentrations of 3, 10 and 30 µM, are 41.4 Hz (95% CI, 36.1–46.5) vs. 33.6 Hz (95% CI, 29.0–38.2) vs. 20.9 Hz (95% CI, 17.1–24.7) vs. 17.4 Hz (95% CI, 14.5–20.3), respectively. There were statistically significant differences in frequency relative to groups (*p* < 0.001). Exact post hoc tests showed that the frequency value was significantly higher in the control group relative to concentrations of 10 µM (*p* < 0.000001) and 30 µM (*p* < 0.000001). Furthermore, the frequency was significantly higher in the 3 µM concentration group relative to the 10 µM (*p* < 0.00001) and 30 µM (*p* < 0.00001) concentrations. There was no statistically significant difference in the frequency of the control compared to the 3 µM concentration (*p* = 0.3442) or the 10 µM concentration compared to the 30 µM concentration (*p* = 1.00) (Figure 4E).

Detailed statistical analysis of frequency among groups in the period before LTP induction showed no statistically significant differences (*p* > 0.05), while statistically significant differences (*p* < 0.0001) were obtained in the induction period and after LTP. During the induction period with a frequency of 100 Hz, it was obtained that the frequency value was significantly higher in the control group compared to the concentrations of 10 µM (*p* = 0.0005) and 30 µM (*p* < 0.000001). Furthermore, the frequency was significantly higher in the 3 µM concentration group relative to the 30 µM concentration (*p* < 0.0001). There was no statistically significant difference in the frequency of the control compared to the 3 µM concentration (*p* = 0.5129), the 3 µM concentration compared to the 10 µM concentration (*p* = 0.1079) and the 10 µM concentration compared to the 30 µM concentration (*p* = 0.1147). In the period after 100 Hz induction, the results were identical to the total value (Figure 4E).

##### Mild Degree of Excitotoxicity

The mean values and 95% CI for ISI in the mild excitotoxicity model and memantine concentrations of 3, 10 and 30 µM were 0.066 (95% CI, 0.061–0.070) vs. 0.045 (95% CI, 0.041–0.048) vs. 0.060 (95% CI, 0.057–0.064) vs. 0.067 (95% CI, 0.064–0.070), respectively. There were statistically significant ISI differences between groups (*p* < 0.0001). Exact post hoc tests showed that in the mild excitotoxicity model group, the ISI value was significantly higher relative to 3 µM concentrations (*p* < 0.000001). Furthermore, ISI was significantly lower in the 3 µM concentration group relative to the 10 µM (*p* < 0.000001) and 30 µM (*p* < 0.000001) concentrations. There was no statistically significant difference in the ISI of the mild excitotoxicity model between the 10 µM concentration (*p* = 0.2152) and the 30 µM concentration (*p* = 1.00) or the 10 µM concentration and the 30 µM concentration (*p* = 0.1259) (Figure 3C). Detailed statistical analysis of ISI against groups in the period before LTP induction showed no statistically significant differences (*p* = 0.4073), while statistically significant differences were obtained in the induction period and after LTP (*p* < 0.0001). During the 100 Hz induction period, it was obtained that the ISI value was significantly higher in the mild excitotoxicity group relative to the 3 µM concentration (*p* = 0.0001). In addition, ISI was significantly higher in the 30 µM concentration group relative to the 3 µM concentration (*p* < 0.000001) and 10 µM concentration (*p* = 0.0013). There was no statistically significant difference in the ISI of mild excitotoxicity between the 10 µM concentration (*p* = 0.0642) and 30 µM concentration (*p* = 1.00) or between the 3 µM concentration and the 10 µM concentration (*p* = 0.2941). In the period after 100 Hz induction, the results were identical to the total value (Figure 3C).

The mean values and 95% CI for EPSP in the mild excitotoxicity group and memantine concentrations of 3, 10 and 30 µM are −43.1 mV (95% CI, −44.9–41.3) vs. −45.5 mV (95% CI, −47.1–43.8) vs. −53.1 mV (95% CI, −54.1–52.1) vs. −54.8 mV (95% CI, −55.5–54.2), respectively. There were statistically significant differences in EPSP between groups (*p* < 0.001). Exact post hoc tests showed that in the mild excitotoxicity group, EPSP was significantly higher relative to concentrations of 3 µM (*p* = 0.0202), 10 µM (*p* < 0.000001) and 30 µM (*p* < 0.000001). In addition, EPSP was significantly higher in the 3 µM concentration group relative to the 10 µM (*p* < 0.000001) and 30 µM (*p* < 0.000001) concentrations. There was no statistically significant difference between the EPSP of the 10 µM concentration and the 30 µM concentration (*p* = 0.8506) (Figure 4B).

Detailed statistical analysis of EPSP among the groups in the period before 100 Hz induction showed no statistically significant differences (*p* > 0.05), while statistically significant differences (*p* < 0.0001) were obtained in the induction period and after 100 Hz. During the 100 Hz induction period, it was obtained that the EPSP value was significantly higher in the mild excitotoxicity group relative to the 10 µM (*p* = 0.0032) and 30 µM (*p* = 0.0001) concentrations. Furthermore, EPSP was significantly higher in the 3 µM concentration group relative to the 10 µM (*p* < 0.000001) and 30 µM (*p* < 0.000001) concentrations. There was no statistically significant difference in EPSP of mild excitotoxicity between the 3 µM concentration (*p* = 1.00), 10 µM concentration and 30 µM concentration (*p* = 0.5771). In the post-induction 100 Hz period, the results were in line with those obtained for the combined analyses (Figure 4B).

The mean values and 95% CI for frequencies in the mild excitotoxicity group and memantine concentrations of 3–30 µM are 23.5 Hz (95% CI, 15.5–28.4) vs. 38.8 Hz (95% CI, 34.0–43.6) vs. 23.9 Hz (95% CI, 19.8–27.9) vs. 17.4 Hz (95% CI, 14.5–20.3), respectively. There were statistically significant differences in frequency relative to groups (*p* < 0.0001). Exact post hoc tests showed that in the mild excitotoxicity group, the frequency value was significantly lower relative to the 3 µM concentrations (*p* < 0.000001). Furthermore, the frequency was significantly higher in the 3 µM concentration group relative to the 10 µM (*p* < 0.00001) and 30 µM (*p* < 0.00001) concentrations. There was no statistically significant difference in the frequency of mild excitotoxicity between the 10 µM (*p* = 0.2152) and 30 µM (*p* = 1.00) concentrations and between the 10 µM concentration and the 30 µM concentration (*p* = 0.1259) (Figure 4F).

Detailed statistical analysis of frequencies among groups in the pre-induction 100 Hz period showed no statistically significant differences (*p* = 0.4073), while statistically significant differences (*p* < 0.0001) were obtained in the induction and post-induction 100 Hz periods. During the 100 Hz induction period, it was obtained that the frequency value was significantly lower in the mild excitotoxicity group relative to the 3 µM concentration (*p* = 0.0001). Furthermore, the frequency was significantly higher in the 3 µM concentration group relative to the 30 µM concentration group (*p* < 0.00001), and significantly higher in the 10 µM concentration group relative to the 30 µM concentration group (*p* = 0.0013). There was no statistically significant difference in the frequency of mild excitotoxicity between the 10 µM concentration (*p* = 0.0642) and 30 µM concentration groups (*p* = 1.00) or the 3 µM concentration and 10 µM concentration groups (*p* = 0.2941). In the period after 100 Hz induction, the results were identical to the total value (Figure 4F).

##### Moderate Degree of Excitotoxicity

The mean values and 95% CI for ISI in the moderate excitotoxicity severity model and memantine concentrations of 3 µM, 10 µM and 30 µM were 0.141 (95% CI, 0.092–0.190) vs. 0.045 (95% CI, 0.041–0.048) vs. 0.053 (95% CI, 0.049–0.057) vs. 0.067 (95% CI, 0.064–0.070), respectively. There were statistically significant ISI differences between groups (*p* < 0.0001). Exact post hoc tests showed that in the medium excitotoxicity model group, the ISI value was significantly higher relative to concentrations of 3 µM (*p* < 0.000001) and 10 µM (*p* = 0.0025). Furthermore, ISI was significantly lower in the 3 µM concentration group relative to the 10 µM (*p* = 0.0150) and 30 µM (*p* < 0.000001) concentrations. A significantly lower ISI value was also obtained in the 10 µM concentration relative to the 30 µM concentration (*p* < 0.000002). There was no statistically significant difference in the ISI of the model of the mean severity of excitotoxicity relative to the 30 µM concentration (*p* = 1.00) (Figure 3E). Detailed statistical analysis of ISI against groups in the period before LTP induction showed no statistically significant differences (*p* = 0.4036), while statistically significant differences were obtained in the induction period and after LTP (*p* < 0.0001). During the 100 Hz induction period, it was obtained that the ISI value was significantly lower in the 3 µM concentration group compared to the 30 µM concentration (*p* < 0.000001). Furthermore, ISI was significantly lower in the 10 µM concentration group relative to the 30 µM concentration (*p* < 0.00001). There was no statistically significant difference in the ISI of the mean intensity of excitotoxicity between the 3 µM (*p* = 0.2186), 10 µM (*p* = 0.9682) and 30 µM (*p* = 0.2540) concentrations or between the 3 µM concentration and the 10 µM concentration (*p* = 1.00). In the post-induction 100 Hz period, the results were identical to the total value and additionally, in the medium excitotoxicity model group, the ISI value was significantly higher relative to 30 µM concentrations (*p* = 0.0002) (Figure 3E).

The mean values and 95% CI for EPSP in the medium excitotoxicity group and memantine concentrations of 3–30 µM are −45.4 mV (95% CI, −48.6–42.2) vs. −44.8 mV (95% CI, −46.6–43.1) vs. −50. mV (95% CI, −51.8–49.4) vs. −54.8 mV (95% CI, −55.5–54.2), respectively. There were statistically significant differences in EPSP among groups (*p* < 0.0001). Exact post hoc tests showed that in the medium excitotoxicity group, the EPSP value was significantly higher relative to the 30 µM concentration (*p* < 0.000001). In addition, EPSP was significantly higher in the 3 µM concentration group relative to the 10 µM concentration (*p* = 0.0017) and 30 µM concentration (*p* < 0.000001) and in the 10 µM group relative to 30 µM (*p* = 0.0031). There was no statistically significant EPSP difference in the mean severity of excitotoxicity between the 3 µM (*p* = 1.00) and 10 µM (*p* = 0.1154) concentrations (Figure 4C).

Detailed statistical analysis of EPSP among groups in the period before 100 Hz induction showed no statistically significant differences (*p* = 0.0828), while statistically significant differences (*p* < 0.0001) were obtained in the induction period and after 100 Hz. During the 100 Hz induction period, it was obtained that the EPSP value was significantly higher in the medium excitotoxicity group relative to the 10 µM (*p* = 0.0058) and 30 µM (*p* = 0.000005) concentrations. In addition, EPSP was significantly higher in the 3 µM concentration group relative to the 10 µM (*p* < 0.000001) and 30 µM (*p* < 0.000001) concentrations and significantly higher in the 10 µM concentration group relative to 30 µM (*p* = 0.0350). There was no statistically significant EPSP difference in the mean intensity of excitotoxicity relative to the 3 µM concentration (*p* = 1.00). In the post-induction 100 Hz period, it was obtained that the EPSP value was significantly higher in the medium intensity excitotoxicity group relative to the 10 µM (*p* = 0.0055) and 30 µM (*p* = 0.0001) concentrations. Furthermore, EPSP was significantly higher in the 3 µM concentration group relative to the 10 µM (*p* = 0.0443) and 30 µM (*p* < 0.000001) concentrations. There was no statistically significant difference in EPSP of the mean intensity of excitotoxicity relative to the 3 µM concentration (*p* = 0.1851) and between the 10 µM concentration and the 30 µM concentration (*p* = 0.0705) (Figure 4C).

The mean values and 95% CI for frequencies in the medium excitotoxicity group and memantine concentrations of 3–30 µM are 26.0 Hz (95% CI, 15.7–36.2) vs. 39.0 Hz (95% CI, 34.2–43.8) vs. 30.9 Hz (95% CI, 26.4–35.4) vs. 17.4 Hz (95% CI, 14.5–20.3), respectively. There were statistically significant differences in frequency between groups (*p* < 0.0001). Exact post hoc tests showed that in the medium excitotoxicity group, the frequency value was significantly lower relative to concentrations of 3 µM (*p* < 0.000001) and 10 µM (*p* = 0.0025). In addition, the frequency was significantly higher in the 3 µM concentration group relative to the 10 µM (*p* = 0.0150) and 30 µM (*p* < 0.00001) concentrations and significantly higher in the 10 µM group relative to the 30 µM group (*p* < 0.00001). There was no statistically significant difference in the frequency of mean intensity of excitotoxicity relative to the 30 µM concentration (*p* = 1.00) (Figure 4G).

Detailed statistical analysis of frequency among the groups in the pre-induction 100 Hz period showed no statistically significant differences (*p* = 0.4036), while statistically significant differences (*p* < 0.0001) were obtained in the induction and post 100 Hz period. During the 100 Hz induction period, it was obtained that the frequency was significantly lower in the 30 µM concentration group compared to the 3 µM concentration (*p* < 0.00001) and 10 µM concentration (*p* < 0.00002). There was no statistically significant difference in frequency of mean excitotoxicity intensity between the 3 µM (*p* = 0.2186), 10 µM (*p* = 0.9682) and 30 µM (*p* = 0.2540) concentrations or between the 3 µM concentration and the 10 µM concentration (*p* = 1.00). In the post-induction 100 Hz period, the results were identical to the total value except that an additional significantly higher frequency was obtained in the 30 µM concentration group relative to the mean excitotoxicity intensity (*p* = 0.0002) (Figure 4G).

##### Advanced Degree of Excitotoxicity

The mean values and 95% CI for ISI in the advanced excitotoxicity severity model and memantine concentrations of 3, 10 and 30 µM were 0.140 (95% CI, 0.093–0.188) vs. 0.046 (95% CI, 0.042–0.049) vs. 0.048 (95% CI, 0.045–0.052) vs. 0.067 (95% CI, 0.064–0.070), respectively. There were statistically significant ISI differences between groups (*p* < 0.001). Exact post hoc tests showed that in the advanced excitotoxicity severity model group, the ISI value was significantly higher relative to concentrations of 3 µM (*p* < 0.000001) and 10 µM (*p* < 0.000001). Furthermore, ISI was significantly higher in the 30 µM concentration group relative to the 3 µM (*p* < 0.000001) and 10 µM (*p* < 0.000001) concentrations. There was no statistically significant difference in the ISI of the advanced excitotoxicity severity model relative to the 30 µM concentration (*p* = 0.1344) and between the 3 µM concentration and the 10 µM concentration (*p* = 1.00) (Figure 3G). Detailed statistical analysis of ISI among groups in the period before LTP induction showed statistically significant differences in the period before and after LTP (*p* < 0.001). No statistically significant differences were obtained in the 100 Hz frequency induction period (*p* = 0.1409). Furthermore, ISI was significantly lower in the 10 µM concentration group relative to the 30 µM concentration (*p* < 0.00001). In the pre-LTP period, the ISI value of advanced excitotoxicity was significantly higher relative to the 3 µM (*p* = 0.0035), 10 µM (*p* = 0.0015) and 30 µM (*p* = 0.0015) concentrations. The other comparisons were not statistically significant (*p* > 0.05). However, in the post-LTP period, the ISI value for the 30 µM concentration was significantly higher relative to that for the 3 µM (*p* < 0.00001) and 10 µM (*p* < 0.00001) concentrations (Figure 3G).

The mean values and 95% CI for EPSP in the advanced excitotoxicity severity group and memantine concentrations of 3–30 µM are −37.8 mV (95% CI, −53.5–22.2) vs. −41.6 mV (95% CI, −44.7–38.6) vs. −44.3 mV (95% CI, −47.1–41.4) vs. −54.8 mV (95% CI, −55.5–54.1), respectively. There were statistically significant differences in EPSP among groups (*p* < 0.0001). Exact post hoc tests showed that in the advanced excitotoxicity severity group, the EPSP value was significantly higher relative to the 30 µM concentration (*p* = 0.0151). In addition, EPSP was significantly higher in the 3 µM concentration group relative to the 30 µM concentration (*p* < 0.000001) and in the 10 µM group relative to 30 µM (*p* < 0.000001). There was no statistically significant difference in the EPSP of advanced excitotoxicity severity between the 3 µM (*p* = 1.00) and 10 µM (*p* = 0.1154) concentrations and between the 3 µM concentration and the 10 µM concentration (Figure 4D).

Detailed statistical analysis of EPSP among the groups in the period before 100 Hz induction showed statistically significant differences (*p* = 0.0063). In the period prior to 100 Hz induction, it was obtained that the EPSP value was significantly higher in the advanced excitotoxicity group relative to concentrations of 3 µM (*p* = 0.0096), 10 µM (*p* = 0.0051) and 30 µM (*p* = 0.0055). There was no statistically significant EPSP difference between the 3 µM concentration and the 10 µM (*p* = 1.00) and 30 µM (*p* = 0.1154) concentrations, or between the 10 µM concentration and the 30 µM concentration (*p* = 1.00). Statistically significant differences were also obtained during the induction period and after 100 Hz (*p* < 0.0001). During the 100 Hz induction period, it was obtained that the EPSP value was significantly higher in the 3 µM concentration group relative to the 30 µM concentration (*p* < 0.000001). Furthermore, EPSP was significantly higher in the 10 µM concentration group relative to the 30 µM concentration (*p* < 0.00001). There was no statistically significant difference in EPSP of the 3 µM and 10 µM concentrations (*p* = 0.5632). In the period after 100 Hz induction, it was obtained that the EPSP value was significantly lower in the 30 µM concentration group relative to the 3 µM (*p* < 0.000001) and 10 µM (*p* < 0.000001) concentrations. There was no statistically significant difference in EPSP of the 3 µM and 10 µM concentrations (*p* = 0.8576) (Figure 4D).

The mean values and 95% CI for frequency in the advanced excitotoxicity severity group and memantine concentrations of 3–30 µM are 9.7 Hz (95% CI, 4.9–14.5) vs. 33.3 Hz (95% CI, 29.3–37.2) vs. 31.8 Hz (95% CI, 27.7–35.9) vs. 17.4 Hz (95% CI, 14.5–20.3), respectively. There were statistically significant differences in frequency relative to groups (*p* < 0.001). Exact post hoc tests showed that in the advanced excitotoxicity group, the frequency value was significantly lower relative to concentrations of 3 µM (*p* < 0.000001) and 10 µM (*p* < 0.000001). Furthermore, the frequency was significantly lower in the 30 µM concentration group relative to the 3 µM (*p* < 0.000001) and 10 µM (*p* < 0.00001) concentrations. There was no statistically significant difference in the frequency of advanced excitotoxicity severity between the 30 µM concentration (*p* = 0.1344) and the 3 µM and 10 µM concentrations (*p* = 1.00) (Figure 4H).

Detailed statistical analysis of frequency among the groups in the pre-induction 100 Hz period showed statistically significant differences (*p* < 0.0001). In the period prior to 100 Hz induction, it was obtained that the frequency value was significantly lower in the advanced excitotoxicity severity group relative to concentrations of 3 µM (*p* = 0.0035), 10 µM (*p* = 0.0015) and 30 µM (*p* = 0.0015). There was no statistically significant difference in the frequency of the 3 µM concentration relative to the 10 µM (*p* = 1.00) and 30 µM (*p* = 1.00) concentrations, or the 10 µM concentration relative to the 30 µM concentration (*p* = 1.00). No statistically significant differences were obtained during the 100 Hz induction period (*p* = 0.1409). In the period after the 100 Hz induction, a significant lower frequency value was obtained in the 30 µM concentration group relative to the 3 µM concentration (*p* < 0.000001) and 10 µM concentration (*p* < 0.000001). There was no statistically significant difference in the frequency of the 3 µM and 10 µM concentrations (*p* = 0.7104) (Figure 4H).

### 3.3. Memory (LTP) and LTP Duration

The results of the simulation studies were statistically analyzed in terms of LTP and LTP duration as described in section “2.2 Long-term synaptic potentiation (LTP) and LTP duration”.

#### 3.3.1. Control Group

**Figure 5:**
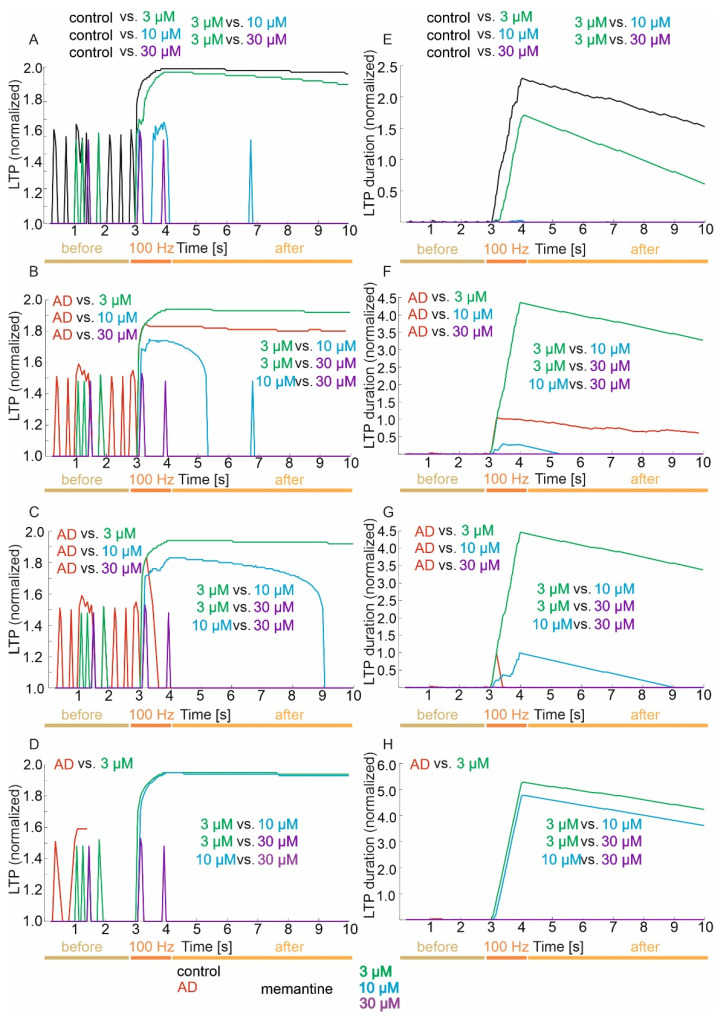
Comparison of memory (LTP) and LTP duration in virtual therapy treatment of memantine treatment at three concentrations: 3, 10 and 30 µM. (**A**,**E**) Control group, (**B**,**F**) mild degree of excitotoxicity, (**C**,**G**) moderate degree of excitotoxicity, and (**D**,**H**) advanced degree of excitotoxicity.

The mean values and 95% CI for LTP in the control group and memantine concentrations of 3, 10 and 30 µM are 1.73 (95% CI, 1.69–1.77) vs. 1.66 (95% CI, 1.62–1.71) vs. 1.07 (95% CI, 1.04–1.10) vs. 1.01 (95% CI, 1.00–1.03), respectively. There were statistically significant differences in LTP versus groups (*p* < 0.001). Exact post hoc tests showed that LTP was significantly higher in the control group relative to concentrations of 3 µM (*p* < 0.000001), 10 µM (*p* < 0.000001) and 30 µM (*p* < 0.000001). Furthermore, LTP was significantly higher in the 3 µM concentration group relative to the 10 µM (*p* < 0.000001) and 30 µM (*p* < 0.000001) concentrations. There was no statistically significant difference in LTP of the 10 µM concentration relative to the 30 µM concentration (*p* = 1.00) (Figure 5A).

Detailed statistical analysis of LTP against groups in the three periods showed statistically significant differences (*p* < 0.0001). In the period prior to 100 Hz induction, it was obtained that LTP was significantly higher in the control group compared to concentrations of 10 µM (*p* = 0.0132) and 30 µM (*p* = 0.0132). There was no statistically significant difference in LTP between the control and the 3 µM concentration (*p* = 0.1296), between the 3 µM concentration and the 10 µM and 30 µM concentrations (*p* = 1.00), and between the 10 µM concentration and the 30 µM concentration (*p* = 1.00).

During the 100 Hz frequency induction period, it was obtained that LTP was significantly higher in the control group relative to 3 µM (*p* = 0.0015) 10 µM (*p* < 0.000001) and 30 µM (*p* < 0.000001) concentrations. Furthermore, LTP was significantly higher in the 3 µM concentration group relative to the 10 µM (*p* = 0.0001) and 30 µM (*p* < 0.000003) concentrations. There was no statistically significant difference in the LTP of the 10 µM concentration relative to the 30 µM concentration (*p* = 1.00). In the period after the 100 Hz induction, the results were identical to the total value (Figure 5A).

The mean values and 95% CI for LTP duration in the control group and memantine concentrations of 3, 10 and 30 µM are 1.410 (95% CI, 1.302–1.517) vs. 0.832 (95% CI, 0.753–0.912) vs. 0.002 (95% CI, 0.00–0.003) vs. 0.0001 (95% CI, 0.000–0.0005), respectively. There were statistically significant differences in LTP duration between groups (*p* < 0.001). Exact post hoc tests showed that the LTP duration value was significantly higher in the control group compared to concentrations of 3 µM (*p* < 0.000001), 10 µM (*p* < 0.000001) and 30 µM (*p* < 0.000001). Furthermore, the duration of LTP was significantly higher in the 3 µM concentration group relative to the 10 µM (*p* < 0.000001) and 30 µM (*p* < 0.000001) concentrations. There was no statistically significant difference in LTP duration of the 10 µM concentration relative to the 30 µM concentration (*p* = 1.00) (Figure 5E).

Detailed statistical analysis of LTP duration among the groups in the three periods showed statistically significant differences (*p* < 0.0001). In the period before the 100 Hz induction, it was obtained that the LTP duration value was significantly higher in the control group relative to the 10 µM (*p* = 0.0133) and 30 µM (*p* = 0.0133) concentrations. There was no statistically significant difference in LTP duration for the control compared to the 3 µM concentration (*p* = 0.1275), between the 3 µM concentration and the 10 µM and 30 µM concentrations (*p* = 1.00), and between the 10 µM concentration and the 30 µM concentration (*p* = 1.00).

During the 100 Hz frequency induction period, it was obtained that the LTP duration value was significantly higher in the control group compared to the concentrations of 3 µM (*p* = 0.0013), 10 µM (*p* < 0.000001) and 30 µM (*p* < 0.000001). Furthermore, the duration of LTP was significantly higher in the 3 µM concentration group relative to the 10 µM (*p* = 0.0001) and 30 µM (*p* < 0.000003) concentrations. There was no statistically significant difference in LTP duration of the 10 µM concentration relative to the 30 µM concentration (*p* = 1.00). In the period after 100 Hz induction, the results were identical to the total value (Figure 5E).

#### 3.3.2. Mild Degree of Excitotoxicity

The mean values and 95% CI for LTP in the mild excitotoxicity group and memantine concentrations of 3–30 µM are 1.64 (95% CI, 1.59–1.68) vs. 1.76 (95% CI, 1.71–1.80) vs. 1.22 (95% CI, 1.17–1.27) vs. 1.01 (95% CI, 1.00–1.03), respectively. There were statistically significant differences in LTP among groups (*p* < 0.001). Exact post hoc tests showed that LTP was significantly lower in the mild excitotoxicity group relative to concentrations of 3–30 µM (*p* < 0.000001). Furthermore, LTP was significantly higher in the 3 µM concentration group relative to the 10 and 30 µM concentrations (*p* < 0.000001) and in the 10 µM group relative to 30 µM (*p* = 0.0220) (Figure 5B).

Detailed statistical analysis of LTP against groups in the three periods showed statistically significant differences (*p* < 0.0001). In the period prior to 100 Hz induction, it was obtained that LTP was significantly lower in the mild excitotoxicity group relative to concentrations of 3 µM (*p* = 0.0165), 10 µM (*p* = 0.0010) and 30 µM (*p* = 0.0010). There was no statistically significant difference in LTP for the 3 µM concentration relative to the 10 µM and 30 µM concentrations (*p* = 1.00) and between the 10 µM concentration and the 30 µM concentration (*p* = 1.00).

During the 100 Hz frequency induction period, it was obtained that the LTP value was significantly lower in the mild excitotoxicity group relative to the 3 µM (*p* = 0.0252) and 30 µM (*p* = 0.0001) concentrations. Furthermore, LTP was significantly higher in the 3 µM concentration group relative to the 10 µM and 30 µM concentrations (*p* < 0.000001). There was no statistically significant difference in the LTP of mild excitotoxicity relative to the 10 µM concentration (*p* = 0.0946) and between the 10 µM concentration and 30 µM concentration (*p* = 0.0564). In the post-induction period of 100 Hz, the results were similar to the total, except there were no significant differences between the 10 and 30 µM concentration groups (*p* = 1.00) (Figure 5B).

The mean values and 95% CIs for LTP duration in the mild excitotoxicity group and memantine concentrations of 3–30 µM are 0.530 (95% CI, 0.470–0.590) vs. 2.819 (95% CI, 2.610–3.029) vs. 0.061 (95% CI, 0.045–0.763) vs. 0.0003 (95% CI, 0.0001–0.0005), respectively. There were statistically significant differences in LTP duration between groups (*p* < 0.001). Exact post hoc tests showed that in the mild excitotoxicity group, the value of LTP duration was significantly lower relative to concentrations of 3 µM–30 µM (*p* < 0.000001). Furthermore, LTP duration was significantly higher in the 3 µM concentration group relative to the 10 µM and 30 µM concentrations (*p* < 0.000001) and significantly higher in the 10 µM group relative to the 30 µM group (*p* = 0.0222) (Figure 5F).

Detailed statistical analysis of LTP duration among groups in the three periods showed statistically significant differences (*p* < 0.0001). In the period prior to 100 Hz induction, it was obtained that the LTP duration value was significantly higher in the mild excitotoxicity group relative to the concentrations of 3 µM (*p* = 0.0160), 10 µM (*p* = 0.0010) and 30 µM (*p* = 0.0010). There was no statistically significant difference in the LTP duration of the 3 µM concentration relative to the 10 and 30 µM concentrations (*p* = 1.00) and that of the 10 µM concentration relative to the 30 µM concentration (*p* = 1.00).

During the 100 Hz frequency induction period, it was obtained that the LTP duration value was significantly lower in the mild excitotoxicity group relative to the 3 µM (*p* = 0.0274) and 30 µM (*p* = 0.0001) concentrations. In addition, the duration of LTP was significantly higher in the 3 µM concentration group relative to the 10 µM and 30 µM concentrations (*p* < 0.000001). There was no statistically significant difference in LTP duration of mild excitotoxicity relative to 10 µM concentration (*p* = 0.0879) and between the 10 µM concentration and the 30 µM concentration (*p* = 0.0572). In the post-induction 100 Hz period, the results were similar to those for the total, except there were no significant differences between the 10 µM and 30 µM concentration groups (*p* = 1.00) (Figure 5F).

#### 3.3.3. Moderate Degree of Excitotoxicity

The mean values and 95% CI for LTP in the medium excitotoxicity group and memantine concentrations of 3–30 µM are 1.25 (95% CI, 1.18–1.32) vs. 1.76 (95% CI, 1.71–1.80) vs. 1.53 (95% CI, 1.48–1.59) vs. 1.01 (95% CI, 1.00–1.03), respectively. There were statistically significant differences in LTP between groups (*p* < 0.001). Exact post hoc tests showed that LTP was significantly lower in the medium excitotoxicity group relative to concentrations of 3 µM (*p* < 0.000001), 10 µM (*p* = 0.0030) and 30 µM (*p* = 0.0298). Furthermore, LTP was significantly higher in the 3 µM concentration group relative to the 10 µM and 30 µM concentrations (*p* < 0.01) and in the 10 µM group relative to 30 µM (*p* < 0.000001) (Figure 5C).

Detailed statistical analysis of LTP against groups in the three periods showed statistically significant differences (*p* < 0.0001). In the period prior to 100 Hz induction, it was obtained that LTP was significantly higher in the medium excitotoxicity group relative to concentrations of 3 µM (*p* = 0.0165), 10 µM (*p* = 0.0010) and 30 µM (*p* = 0.0010). There was no statistically significant difference in LTP between the 3 µM concentration and the 10 µM and 30 µM concentrations (*p* = 1.00) or between the 10 µM concentration and the 30 µM concentration (*p* = 1.00).

During the 100 Hz induction period, it was obtained that LTP was significantly lower in the medium excitotoxicity group relative to the 3 µM concentration (*p* = 0.0011). Furthermore, LTP was significantly higher in the 3 µM concentration group relative to the 10 µM and 30 µM concentrations (*p* < 0.000001) and the 10 µM concentration relative to the 30 µM concentration (*p* = 0.0023). There was no statistically significant difference in the LTP of mean excitotoxicity intensity between the 10 µM (*p* = 1.00) and 30 µM (*p* = 0.1157) concentrations. In the post-induction 100 Hz period, the results were similar to those for the total, except there was no significant difference between the groups of mean excitotoxicity severity and 30 µM (*p* = 1.00) (Figure 5C).

The mean values and 95% CI for LTP duration in the medium excitotoxicity group, memantine concentration 3 µM–30 µM are 0.063 (95% CI, 0.014–0.111) vs. 2.906 (95% CI, 2.693–3.118) vs. 0.346 (95% CI, 0.300–0.392) vs. 0.0003 (95% CI, 0.0001–0.0005), respectively. There were statistically significant differences in LTP duration between groups (*p* < 0.001). Exact post hoc tests showed that in the medium excitotoxicity group, the LTP duration value was significantly lower relative to concentrations of 3 µM (*p* < 0.000001), 10 µM (*p* = 0.0030) and 30 µM (*p* = 0.0299). Furthermore, LTP duration was significantly higher in the 3 µM concentration group relative to the 10 µM and 30 µM concentrations (*p* < 0.01) and significantly higher in the 10 µM group relative to the 30 µM group (*p* < 0.000001) (Figure 5G).

Detailed statistical analysis of LTP duration against groups in the three periods showed statistically significant differences (*p* < 0.0001). In the period prior to 100 Hz induction, it was obtained that the LTP duration value was significantly higher in the medium excitotoxicity group compared to concentrations of 3 µM (*p* = 0.0160), 10 µM (*p* = 0.0010) and 30 µM (*p* = 0.0010). There was no statistically significant difference in the LTP duration of the 3 µM concentration relative to the 10 µM and 30 µM concentrations (*p* = 1.00) and the 10 µM concentration relative to the 30 µM concentration (*p* = 1.00).

During the 100 Hz frequency induction period, it was obtained that the LTP duration value was significantly lower in the medium excitotoxicity group against the 3 µM concentrations (*p* = 0.0010). Furthermore, the LTP duration was significantly higher in the 3 µM concentration group relative to the 10 µM and 30 µM concentrations (*p* < 0.000001) and significantly higher in the 10 µM group relative to the 30 µM group (*p* = 0.0024). There was no statistically significant difference in LTP duration of mean excitotoxicity intensity against 10 µM (*p* = 1.00) and 30 µM concentrations (*p* = 0.1225). In the post-induction 100 Hz period, the results were similar to those for the total, except there were no significant differences between the 10 µM and 30 µM concentration groups (*p* = 1.00) (Figure 5G).

#### 3.3.4. Advanced Degree of Excitotoxicity

The mean values and 95% CI for LTP in the advanced excitotoxicity severity group and memantine concentrations of 3–30 µM are 1.32 (95% CI, 1.12–1.53) vs. 1.77 (95% CI, 1.72–1.82) vs. 1.73 (95% CI, 1.68–1.79) vs. 1.01 (95% CI, 1.00–1.03), respectively. There were statistically significant differences in LTP among groups (*p* < 0.001). Exact post hoc tests showed that LTP was significantly lower in the advanced excitotoxicity severity group relative to 3 µM concentrations (*p* = 0.0008). Furthermore, LTP was significantly higher in the 3 µM concentration group relative to the 10 µM and 30 µM concentrations (*p* < 0.00001) and in the 10 µM group relative to 30 µM (*p* < 0.000001) (Figure 5D).

Detailed statistical analysis of LTP against groups in the three periods showed statistically significant differences (*p* < 0.0001). In the period prior to 100 Hz induction, it was obtained that LTP was significantly higher in the advanced excitotoxicity group relative to 10 µM (*p* = 0.0206) and 30 µM (*p* = 0.0206) concentrations. There was no statistically significant difference in LTP of the 3 µM concentration relative to the 10 µM and 30 µM concentrations (*p* = 1.00) or the 10 µM concentration relative to the 30 µM concentration (*p* = 1.00).

During the 100 Hz induction period, it was obtained that LTP was significantly higher in the 3 µM concentration group relative to the 30 µM concentration (*p* < 0.000001) and the 10 µM concentration relative to the 30 µM concentration (*p* = 0.00003). There was no statistically significant difference in LTP of the advanced 3 µM and 10 µM concentrations (*p* = 1.00). In the post-induction 100 Hz period, LTP was significantly higher in the 3 µM group relative to 10 µM and 30 µM concentrations (*p* < 0.01) and in the 10 µM group relative to 30 µM (*p* < 0.01) (Figure 5D).

The mean values and 95% CI for LTP duration in the advanced excitotoxicity severity group and memantine concentrations of 3–30 µM are 0.013 (95% CI, 0.022–0.025) vs. 3.700 (95% CI, 3.435–3.956) vs. 3.176 (95% CI, 2.931–3.421) vs. 0.0003 (95% CI, 0.0001–0.0005), respectively. There were statistically significant differences in LTP duration between groups (*p* < 0.001). Exact post hoc tests showed that the LTP duration value was significantly lower in the advanced excitotoxicity group relative to the 3 µM concentrations (*p* = 0.0006). Furthermore, efficiency was significantly higher in the 3 µM concentration group relative to the 10 µM and 30 µM concentrations (*p* < 0.000001) and significantly higher in the 10 µM group relative to the 30 µM group (*p* < 0.000001) (Figure 5H).

Detailed statistical analysis of LTP duration against groups in the three periods showed statistically significant differences (*p* < 0.0001). In the period prior to 100 Hz induction, it was obtained that the LTP duration value was significantly higher in the advanced excitotoxicity group against 10 µM (*p* = 0.0207) and 30 µM (*p* = 0. 0207) concentrations. There was no statistically significant difference in the LTP duration of the 3 µM concentration relative to the 10 µM and 30 µM concentrations (*p* = 1.00) and the 10 µM concentration against the 30 µM concentration (*p* = 1.00) and advanced excitotoxicity severity relative to the 3 µM concentration (*p* = 0.0839).

During the 100 Hz induction period, it was obtained that the LTP duration value was significantly lower in the 30 µM group relative to the 10 µM (*p* < 0.000001) and 30 µM (*p* = 0.00003) concentrations. There was no statistically significant difference between the 3 µM and 10 µM concentrations (*p* = 1.00). In the period after 100 Hz induction, the LTP duration value was significantly higher in the 3 µM group relative to the 10 µM and 30 µM concentrations (*p* < 0.01) and in the 10 µM group relative to 30 µM (*p* < 0.01) (Figure 5H) (see Supplementary Materials).

## 4. Discussion

Several studies have shown that memantine, when used in doses where it has a neuroprotective effect, also has a beneficial effect on memory. Our computer simulations confirm the biological findings. We obtained no differences in inter-spike intervals between the control model and the model using the lowest dose of memantine. Furthermore, we obtained similar results for frequency and EPSP. Our results are consistent with numerous preclinical studies showing that therapeutic concentrations of memantine (1–3 μM) have neuroprotective effects. They do not cause side effects in learning impairment and long-term synaptic potentiation (LTP). Our study also confirms, as in experiments in healthy volunteers, that memantine at higher doses did not affect cognition [43,44,45].

Substances that inhibit the pathological stimulation of NMDA receptors may improve the physiological function of neurons in the hippocampal area and alleviate the symptoms of dementing diseases. Experiments show that memantine prevents pathological changes in hippocampi [18], and when administered to rats before NMDA injections, it inhibited neuronal death [46]. In our simulation experiments, we confirm that the use of memantine in models of excitotoxicity severity (mild, moderate and very advanced) resulted in shortened ISI and increased LTP, long-term synaptic gain time, frequency and EPSP.

Studies with the use of animal models have shown that both single and chronic administrations of memantine, corresponding to therapeutic doses used in dementia patients, significantly affect NMDA receptors, and that exceeding a dose of 20 mg/kg can cause side effects [18]. The beneficial effects of memantine on the cognitive performance of AD patients have been demonstrated in several multicentre studies that included patients with moderate to profound AD who received memantine monotherapy [47]. In experiments conducted on old animals (2-year-old rats), memantine prolonged LTP and improved the ability to maintain memory traces [43,44,45]. A statistically significant advantage of memantine in monotherapy over placebo was demonstrated [48]. Our simulation experiments confirm that the use of memantine in models of excitotoxicity severity (intermediate and very advanced) as measured by LTP and LTP duration results in a statistically significant increase. In both models, the most effective dose of memantine was 10 µM, which caused a significant reduction in ISI and an increase in frequency.

Occasionally, memantine is prescribed for the treatment of mild AD and cognitive degeneration due to studies that have indicated its efficacy [49]. In our study in a mild model of LTP excitotoxicity, LTP time and frequency increased significantly after 3 µM memantine administration. In the EPSP area, we obtained an increase for all doses of 3–30 µM.

## 5. Conclusions

The NMDA antagonist memantine causes neuroprotective benefits in patients with moderate to severe AD. One of the most important benefits of memantine is the improvement of cognitive function and beneficial effects on memory. On the other hand, memantine provides only symptomatic and temporary support for AD patients. Memantine is prescribed in the US and Europe if a patient has moderate to severe AD. Memantine has also been approved for mild to moderate AD patients. However, its very modest effect provides motivation for further research into new drugs in AD. We are the first to present a mathematical model of the NMDA receptor that allows simulation of excitotoxicity and virtual memantine therapy.

## 6. Limitations

A limitation of the study is any simulation study that relies on a mathematical and computer model. Although our model reflects the mechanisms of both NMDA receptor function and simulation of excitotoxicity severity very well, it will never match the complexity of biological systems.

## Data Availability

All data generated or analysed during this study are included in this published article (and its Supplementary Materials files).

## Supplementary Materials

The following supporting information can be downloaded at: https://www.mdpi.com/article/10.3390/jcm11071858/s1, 1_Spikes—data file numbers of spikes, 2_LTP—data file LTP, 3_LTP duration—data file LTP duration, 4_EPSP—data file EPSP. All folders contain subfolders: 1_control group and 2_models of excitotoxicity severity AD. The folder structure is as follows: Supplementary Materials/[1_Spikes or 2_LTP or 3_LTP duration or 4_EPSP]/[1_control group] or [2_models of excitotoxicity severity AD]/[1_withou memantine] or [2_memantine treatment]/[3M or 10M or 30M]. The program code is available on the website: https://github.com/dswietlik/Dariusz-Swietlik/blob/main/Neuron%20model (accessed on 17 February 2022).
